# Climate change maladaptation for health: Agricultural practice against shifting seasonal rainfall affects snakebite risk for farmers in the tropics

**DOI:** 10.1016/j.isci.2023.105946

**Published:** 2023-01-07

**Authors:** Eyal Goldstein, Joseph J. Erinjery, Gerardo Martin, Anuradhani Kasturiratne, Dileepa Senajith Ediriweera, Ruchira Somaweera, Hithanadura Janaka de Silva, Peter Diggle, David G. Lalloo, Kris A. Murray, Takuya Iwamura

**Affiliations:** 1 School of Zoology, Department of Life Sciences, Tel Aviv University, Tel Aviv, Israel; 2 Ecosystem Modeling, University of Göttingen, Göttingen, Germany; 3 Department of Zoology, Kannur University, Kannur, India; 4 Escuela Nacional de Estudios Superiores unidad Mérida, Universidad Nacional Autónoma de México, Yucatán, México; 5 Department of Public Health, Faculty of Medicine, University of Kelaniya, Kelaniya, Sri Lanka; 6 Health Data Science Unit, Faculty of Medicine, University of Kelaniya, Ragama, Sri Lanka; 7 School of Biological Sciences, University of Western Australia, Perth, WA, Australia; 8 Deparment of Medicine, Faculty of Medicine, University of Kelaniya, Ragama, Sri Lanka; 9 CHICAS, Lancaster University Medical School, Lancaster, UK; 10 Johns Hopkins Bloomberg School of Public Health, Baltimore, MD, USA; 11 Liverpool School of Tropical Medicine, Liverpool, United Kingdom; 12 Centre on Climate Change and Planetary Health, MRC Unit the Gambia at London School of Hygiene and Tropical Medicine, Fajara, The Gambia; 13 Department F.-A. Forel for Aquatic and Environmental Science, University of Geneva, Geneva, Switzerland

**Keywords:** Agricultural science, Applied sciences, Food science, Sustainability aspects of food production

## Abstract

Snakebite affects more than 1.8 million people annually. Factors explaining snakebite variability include farmers’ behaviors, snake ecology and climate. One unstudied issue is how farmers’ adaptation to novel climates affect their health. Here we examined potential impacts of adaptation on snakebite using individual-based simulations, focusing on strategies meant to counteract major crop yield decline because of changing rainfall in Sri Lanka. For rubber cropping, adaptation led to a 33% increase in snakebite incidence per farmer work hour because of work during risky months, but a 17% decrease in total annual snakebites because of decreased labor in plantations overall. Rice farming adaptation decreased snakebites by 16%, because of shifting labor towards safer months, whereas tea adaptation led to a general increase. These results indicate that adaptation could have both a positive and negative effect, potentially intensified by ENSO. Our research highlights the need for assessing adaptation strategies for potential health maladaptations.

## Highlights

• Climate change adaptation strategies can lead to unexpected snakebite risk
• ENSO has a significant effect on risk depending on season and direction of ENSO extreme
• Some strategies may lower aggregate snakebites but increase risk per individual farmer

## Graphical abstract

**Figure:**
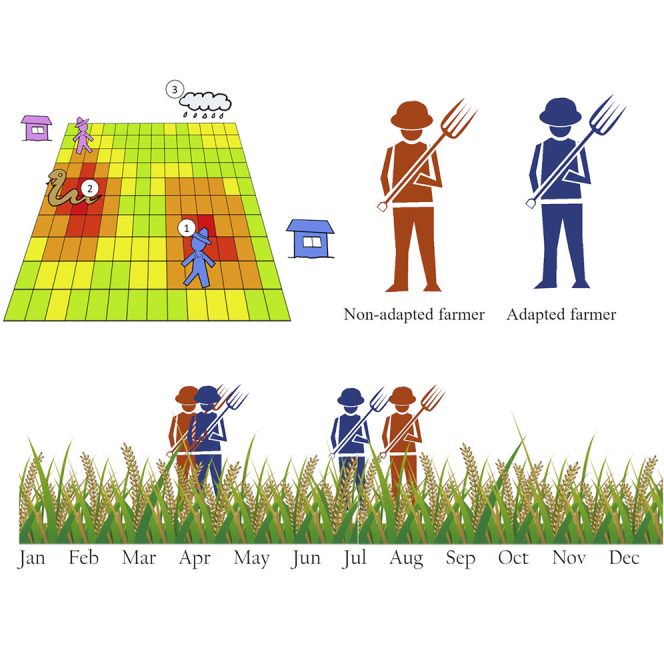

## Introduction

Climate change has caused large-scale social and ecological impacts, affecting multiple aspects of life on earth.^1^ In response, societies are increasingly adapting or planning adaptation strategies to reduce the risks of a changing climate. Where such responses designed to protect infrastructure, livelihoods or well-being inadvertently increase other risks, such as the direct or indirect health impacts of climate change, climate maladaptation may result. Clearly, it is necessary to evaluate the potential risks of climate change adaptation strategies alongside the intended benefits, particularly where these may harm human health.

Snakebite is climate-sensitive neglected tropical disease (NTD) particularly affecting rural farmers and communities living in tropical areas,^2^ with up to 1.8 million bites and up to 94,000 deaths annually.^2^^,^^3^ Rural farmers and communities in the tropics are also on the frontline of climate change impacts, and are increasingly adopting strategies to reduce the impacts of a changing climate on their livelihoods, health and well-being. These strategies may target protecting crop yields in the face of rising temperatures and changing patterns of precipitation, which could include switching to more climate-resilient crop types, changing crop rotation schedules, or changing daily activity patterns. Given snakebite is driven by complex social, ecological, and economic interactions,^4^ such climate change adaptation strategies have clear potential to directly influence farmer exposure to snakes and subsequently snakebite risk. Understanding the potential impact of farmer adaptation strategies on snakebite risk is therefore an important but currently overlooked component of meeting the targets of the WHO snakebite roadmap to 2030.^5^^,^^6^

Existing studies highlight both social and ecological factors influencing snakebite incidence, including occupational and behavioral traits of affected populations alongside climate and other factors related to snake ecology. Different models have, for example, estimated the contribution of climatic factors,^4^ venomous snake distributions,^7^ and extreme weather events to snakebite risk.^8^^,^^9^^,^^10^ In addition, models have predicted changes in the risk of snakebite because of climate change, as a result of changes in weather patterns^11^ and changes in venomous snake distributions.^12^ These studies are consistent with many others that show that changes in temperature and precipitation patterns and the frequency or magnitude of extreme climate events such as flood or droughts commonly influence the distribution, abundance and behavior of disease causing species, thereby influencing the distribution and burden of many human diseases.^13^ For this reason, natural variations in climate, such as those associated with the seasonal movement of the Intertropical Convergence Zone (ITCZ) or the multiyear cycles of El Nino Southern Oscillation (ENSO) are also often also linked to changing patterns of human disease risks. Some of the most significant effects of climate change on human health are linked to its numerous influences on these natural climate cycles; for instance, intensifying ENSO cycles in the tropics has direct implications for climate-sensitive infectious diseases^14^ as well as snakebite.^4^

On the socio-economic side, changes in climate and extreme weather events also induce societal changes, such as farmers’ adaptation of new behaviors and activity patterns. In some cases, these changes may similarly increase exposure to disease causing species; for example, by increasing the frequency of human-wildlife encounters.^15^ In response to climate change, farmers are already trying to mitigate the potential harms of shifting climatic patterns to agriculture with adaptive behaviours.^16^ Such adaptation strategies used by farmers include: changes in seasonal patterns of planting because of altered monsoon onset, changes in crop varieties because of increased risk of drought, changing allocation of labor between seasons and hours of the day because of altered and extreme weather patterns, and use of new technologies.^17^^,^^18^ So far, however, the extent to which climate change adaptation may affect snakebite risk remains unstudied.

Here, we developed a framework to explore how such climate change adaptation strategies could affect snakebite patterns on the island nation of Sri Lanka, a snakebite hotspot with an estimated >30,000 envenomings and 400 deaths by snakebite annually.^19^ In Sri Lanka, spatial and temporal patterns of snakebite correlate with climatic conditions.^11^ Sri Lankan farmers are particularly vulnerable to snakebites,^36^ and temporal peaks of snakebite incidence coincide with peak subsistence agricultural activities, such as rice harvest.^11^ Snakebite incidence can also partially be explained by the predicted abundance, distribution and behavioral traits of the key species venomous snakes.^20^ Simultaneously, Sri Lankan farmers are also highly vulnerable to climate change as well as changes in ENSO because of climate change,^17^^,^^21^^,^^22^ which are forcing farmers to adapt accordingly. Adaptation strategies used in Sri Lanka include shifting rice planting patterns because of delayed monsoon,^17^^,^^23^ changes in the allocation of labor in tea plantations as a result of changing rainfall patterns,^21^^,^^24^ and the introduction of new rubber harvesting methods that are better suited to drought conditions.^25^

When modeling future snakebite burden under climate change, previous research has relied heavily on empirical statistical analysis,^11^^,^^26^ which has limited value for representing the diversity in individual farmers’ behaviors and pinpointing mechanisms that underlie changes in contact between humans and snakes, a fundamental requirement for snakebite to occur in the first place. The present study explores the implications of climate change adaptation strategies on farmers’ risk of snakebite using mechanistic agent based models, parameterized with field data, to simulate snake-human contact and predict patterns of snakebite risk.^57^ To our knowledge this is the first study to examine the effect of farmers’ individual uptake of climate adaptation strategies on snakebite risk under different climatic conditions induced by ENSO. We interpret increases in snakebite incidence in the three major agricultural crops in Sri Lanka (rubber, tea and rice) as examples where changes in agricultural practices to manage climate change risks come at the cost of unexpected human health risks, an example of ‘maladaptation’.^27^^,^^28^ This modeling effort could further help mitigate snakebites and assist in the WHO plan to reduce snakebites mortality by 50% by the year 2030.^29^

## Methods

### Study area

Sri Lanka is divided into four different climatic regions characterized by their precipitation levels.^30^ Sri Lanka has two monsoonal systems that are highly influenced by El Nino and La Nina phases,^40^ and exhibits high rainfall variability^34^^,^^40^ as well as recurring droughts in both dry and wet regions.^31^ Although climate change predictions are highly variable between different regions and altitudes across the island, generally they can be characterized by delay in the timing of monsoonal onset,^23^ as well as increased frequency of extreme rain and drought events.^30^^,^^31^

We used the district of Ratnapura, located in the wet and intermediate zones of Sri Lanka, as our case study, both because of its high diversity of crops (tea, rubber, rice, and coconut),^32^^,^^33^ and because it has consistently stable mean daily temperatures which fluctuates between 27-29 ° C all year around. This climatic stability allowed us to disentangle the compounding effect of rainfall and mean temperature on the propensity of snakes to bite. The Ratnapura region also contains some of the most venomous snakes in Sri Lanka, with three different medically important snakes present – *Daboia russelli*, *Naja naja* and *Hypnale hypnale*^35^ – and is a major hotspot for snakebites on the Island.^36^ In addition, Ratnapura district is experiencing rapid changes in crop types, crop varieties and the agricultural calendar as farming communities try to adapt to a changing climate.^17^ Although Ratnapura has both large scale and small scale plantations, our study focused on small scale farmers. For landcover maps used in the model see Supplementary material 1.

### Model description

Agent based modeling (ABM) is a bottom up approach that simulates behavioural traits of individual agents, and their interactions with one another and the environment.^37^ Our simulations rely on an ABM that was previously described at Goldstein et al., 2021,^57^ which simulated human-snake interactions based on a comprehensive dataset from Sri Lanka. The model includes information on estimated snake abundance, behavioral traits relevant to snakebite, landcover preferences for different biting species,^20^ and farmer seasonal and daily activity patterns (see Table 1). In addition, a landcover classification derived from remote sensing data provides data on the different landcover categories present in the region, which include rice, rubber, tea, forest, and water bodies.

**Table 1:** Brief description of variables used in the model (adapted from Goldstein et al. 2021)

Model unit	Parameter	Value
Farmers	Farmertype	Rice, Rubber, Tea
Farmers	Landtype work index	0–110
Farmers	Starting hour	4–9AM
Farmers	Number of hours worked	4–14
Snake	Point process models	0–3∗10ˆ^−8^
Snake	Seasonal activity probability	0–1
Snake	Daily activity patterns	0–1
Snake	Aggressiveness	1–10
Snake	Land association factor	0–2.429
Land cover	Type of land cover	Rice, Tea, Rubber, Forest, Water, Home
Climate	Mean monthly precipitation	0–1500
Climate	Number of rainy days	0–31

Our model includes two types of agents: farmers and snakes. Each farmer is an autonomous agent who works on a number of possible agricultural land cover types according to the scenario that is ascribed. Farmers have working schedules that include the land cover type they should be farming, time of day they begin to work, and the number of hours they will spend working in that land cover class. Using the work schedule, based on information gathered during field work, the farmers move between the land cover they are farming and their home.

Each snake agent is characterized by a set of ecological and behavioral traits, including: species, daily activity, habitat preference, propensity to bite, and seasonal activeness. Each species is given a set of probabilities of moving between land cover classes depending on their habitat preferences and the area covered by each land cover class. Seasonal activity of each snake species in the model is driven by precipitation patterns.

Simulations took place over the period 2008–2017 to capture the longer-term interannual variation in climate because of ENSO, which sees multiyear oscillations between El Nino and La Nina conditions. This period included the El Nino events of both 2009–2010 & 2014–2016. Although ENSO events can have varying outcomes in precipitation timing and levels across different regions within Sri Lanka,^38^ for the country as a whole the patterns can be summarized as follows: January–March both El Nino and La Nina decreases precipitation, April–June El Nino increases precipitation and La Nina decreases it, July-August La Nina increases precipitation and El Nino decreases it, October–December El Nino increases precipitation whereas La Nina decreases it.^39^^,^^40^

**Figure 1:**
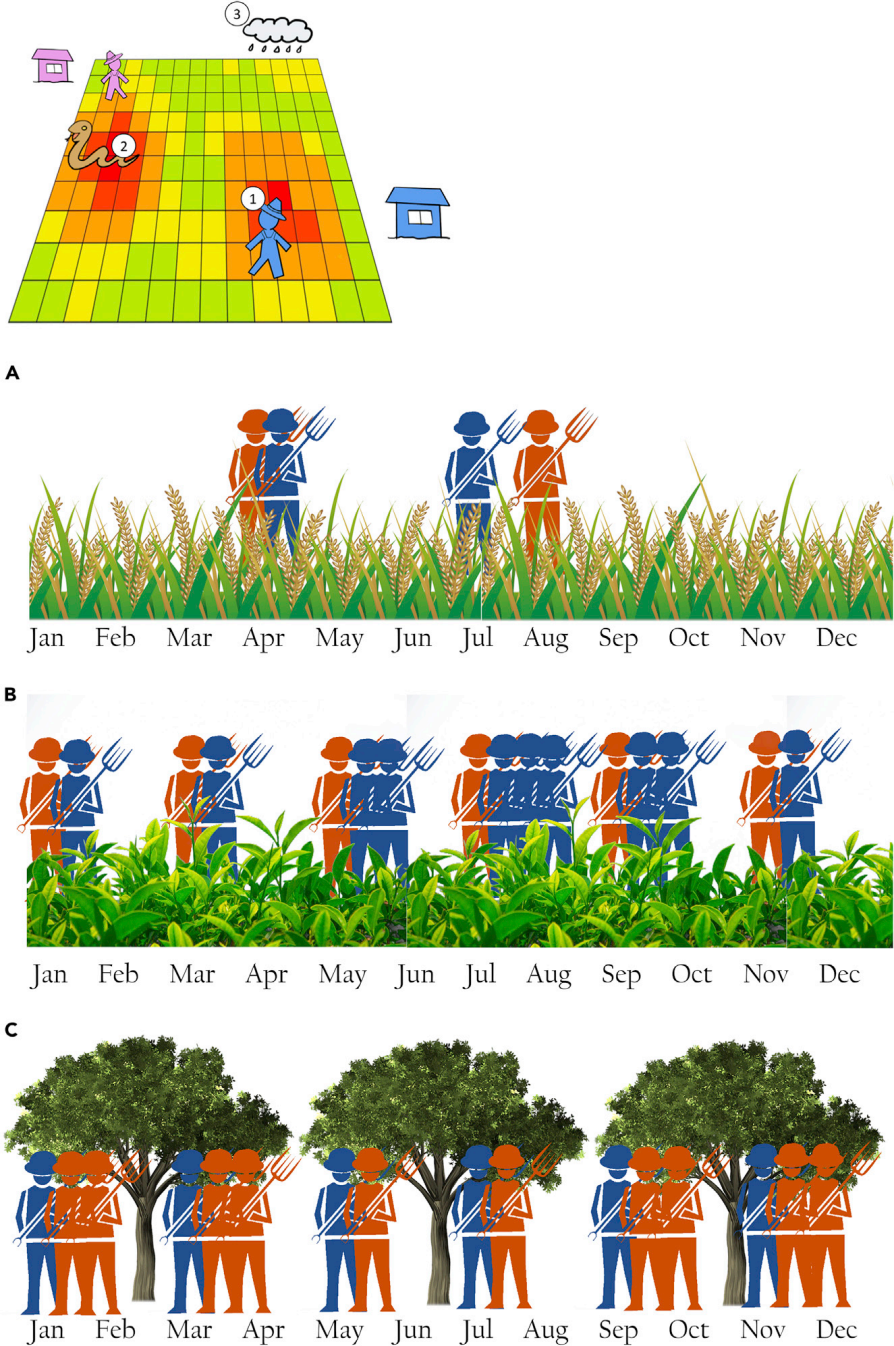
Farmers (1) and snakes (2) both respond to precipitation (3), but as farmers adapt their strategies to climate change, their responses change, and as a result the pattern of contacts between snakes and farmers change (A) Rice farmers using adapted strategies to climate change choose to plant short growth rice. (B) Tea farmers using adapted strategies to climate change choose to utilize labor during different times to optimize harvest. (C) Rubber farmers using adapted strategies to climate change harvest rubber using a low-frequency tapping methods instead of harvesting during all non-rainy days.

The purpose of the model is to represent the spatio-temporal overlap between farmers and snakes according to the prevailing weather (2008–2017), with the farmers reacting to longer term climatic and landscape conditions by adapting their farming practices. Both farmers and snakes seasonally interact with their climate and landscape, which defines their behavioral patterns, and when they meet while being active, the model tracks the possibility of a snakebite occurring according to the different species’ propensities to bite.^20^ We then run the model according to different climate adaptation scenarios for each crop type (detailed below), and follow the snakebite outcomes accordingly. For each scenario, we compared the outcomes of the no adaptation (baseline) versus adaptation strategies across all years (Figure 1).

The model has been previously validated using multiple patterns on which there was already research conducted in Sri Lanka, such as seasonal patterns of snakebites,^11^ the relative risk of snakebite between locations,^36^ and biting snake species composition among bite victims as inferred from hospital records across Sri Lanka^19^(see Goldstein et al. 2021). This validation was done in accordance with the Pattern oriented modeling protocol (POM),^41^ which suggests that multiple patterns be assessed and the fit between the model predictions and these patterns evaluated (as opposed to comparing results to a single statistic or a single pattern). This is supposed to prevent overfitting of the model to an expected output, or falsely representing the model by using only one output parameter, and to make sure that the model can represent the dynamics of the system that it is attempting to represent.

### Adaptation scenarios

Although there are multiple adaptation strategies for each crop type, and each one of these adaptations may affect snakebite outcome in different ways, our focus was on those adaptation strategies that have already been suggested within the peer-reviewed published literature as appropriate for the region, as well as those that involve farmers modifying their temporal (daily, monthly, seasonally) presence in agricultural fields. Although our set of scenarios did not include all possible adaptations, they did include an important subset of adaptations that are currently in the scope of farmers dealing with climate change in Sri Lanka as well as elsewhere, including: change in rice crop variety (hereafter “Rice”), change in mechanization that leads to altered routine in rubber harvesting ("Rubber”), and change in harvest intensity of tea (“Tea”).

#### Rice

Owing to changes in precipitation patterns, farmers may change their rice cultivation practices to sustain productivity.^17^^,^^42^ For instance, previous research in the Kurunegala district of Sri Lanka has shown that shifting forward planting dates in response to drought can have a positive effect on rice yield,^42^ whereas other research has highlighted the importance of changing rice varieties to adapt to greater climate variability.^43^ Sri Lanka has a large diversity of rice varieties, ranging in growth period from two to six months.^44^ In addition, Sri Lanka has two rice growing periods, Maha and Yala. Although the most popular rice variety in the region is BG 352 because of its higher productivity, it requires between 3-4 months to cultivate and is susceptible to drought events.^44^ As a climate adaptation strategy to cope with earlier arrival of the dry season, and changing monsoon dates, farmers are increasingly using plant varieties with shorter growth periods, such as BG 750 which requires only 2 ½ months for full maturation but has a lower yield per annual cycle.^45^ With precipitation patterns predicted to shift in the future, more farmers are expected to shift from long to short-duration paddy variety, which may change their exposure to the risk of snakebite.^17^

In this scenario, we tested whether the use of a short-duration paddy variety would affect snakebite risk. Farmers in the simulation are allowed to plant either a conventional variety with three months growth period and harvest in the fourth month, or a drought-resistant variety with two months growth period and harvest at the third month. We focus on the differences in snakebite risk under years with different precipitation patterns that reflect the impacts of ENSO.

#### Rubber

Traditional rubber extraction methods require farmers to harvest on all non-rainy days, because rain mixing with sap during harvesting can contaminate and degrade the product.^46^ Rubber trees are, however, sensitive to over-tapping whereas drought conditions reduce tap yields.^47^ Furthermore, the combination of over-tapping and drought conditions can increase the chance of tapping panel dryness,^48^^,^^49^ which can result in complete crop loss. An adaptation strategy available to farmers is to use a low frequency tapping method, which allows them to harvest more continuously throughout the year. This method is more resilient to climate change as it moderates extraction and reduces the risk of over-tapping and disease during low-rainfall periods.^25^ Although this method is more costly because of the need for special equipment used to protect sap from rainfall, with increasing frequency and magnitude of drought predicted in Sri Lanka it is expected to become a viable climate adaptation strategy for rubber farmers.

We thus created scenarios with different rubber-tapping methods in which we compared snakebite risk between the current practice of farmers continuously tapping rubber trees on non-rainy days (see Goldstein et al., 2021) with rainy days as defined by Domroes and Ranatunge 1993,^52^ leading to approximately 150 workdays per year. For farmers implementing the adaptation strategy of low frequency tapping, rubber trees are tapped only once every five days irrespective of rainfall, through the entire year.

#### Tea

The intensity of tea harvesting in Sri Lanka shows a bimodal annual pattern, where peaks occur one month after the peaks of precipitation associated with the two monsoon seasons.^50^ Tea productivity is positively correlated with rainfall in the previous month.^24^ It has been suggested that many farmers do not currently exploit these tea yield peaks because of lack of responsiveness in labor supply.^51^ Climate change is predicted to exacerbate tea production issues in future. For instance, recent models have predicted that both global and Sri Lankan tea productivity under certain climate scenarios could decline because of increased climate variability, whereas demand continues to rise.^53^

Tea farmers could adapt to climate change-induced tea production uncertainties by better exploiting the rain-yield relationship to optimize labor supply with respect to peak harvest periods. However, this could change their exposure to snakebite, which is also strongly linked to rainfall events and seasonality. As such, we developed a tea-harvest scenario in which we compared current tea harvesting strategies (harvest is consistent during the year; see Goldstein et al., 2021). We used an optimized tea harvest strategy that predicts and meets labor requirements on the basis of the linear relationship between yield and rainfall in the month before optimal rainfall, with a work increase of 0.514 labor hours per hectare above the baseline for every mm of rainfall.^24^^,^^50^

### Simulation output analysis

Our simulations tracked a list of different outputs, including location, time, and snake species causing snakebites (previously described in Goldstein et al. 2021). These outputs were later used to understand the contribution of the different input variables (scenario design and climate) to the final snakebite patterns. Finally we used the extraneous El Nino index to understand how global climate patterns affect local snakebites.

For each of the scenarios, we compared the mean number of snakebites of the current farming practice to the alternative climate change adaptation strategy with nonparametric Mann-Whitney U tests. We compared the results both for the total number of snakebites, as well as for snakebites divided by number of hours spent in fields, which represents incidence rate per farmer per hour. The sample size for each group was 5,100 simulation points.

We then conducted a linear regression analysis for the different adaptation strategies to assess the association between on the number of snakebites and the monthly oceanic Nino index (ONI), which is based on sea surface temperature of the Nino 3.4 region (taken from noaa.gov). For this analysis we divided the year into the four different seasons in Sri Lanka (North east monsoon, first intermonsoon, South west monsoon, and second intermonsoon), and analysed the relationship between the index and the number of snake bites for each season period, because each season is affected differently by the different phases of the ENSO cycle.

## Results

### Rice

**Figure 2:**
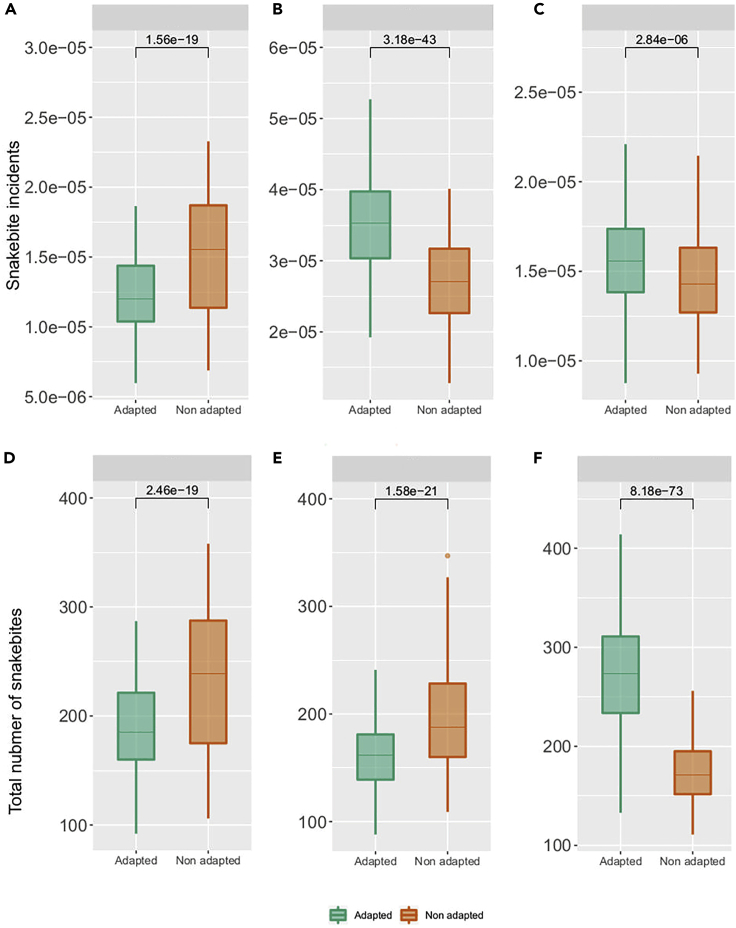
Difference in number of snakebites between adapted and non-adapted farmers normalized by the number of hours spent in the field to represent snakebite incidence per farmer per hour (A) For rice farmers adapting significantly decreases snakebite risk per farmer. (B) For rubber farmers adapting significantly decreases snakebite risk per farmer. (C) For tea farmers adapting significantly increase in snakebite risk per farmer. Difference in total yearly number of snakebites between adapted and non-adapted farmers representing the total number of snakebites caused by adaptation. (D) For rice farmers adapting leads to a significant decrease in snakebites. (E) For rubber farmers adapting leads to a significant increase of snakebites. (F) For tea farmers adapting causes a significant increase of snakebites.

**Figure 3:**
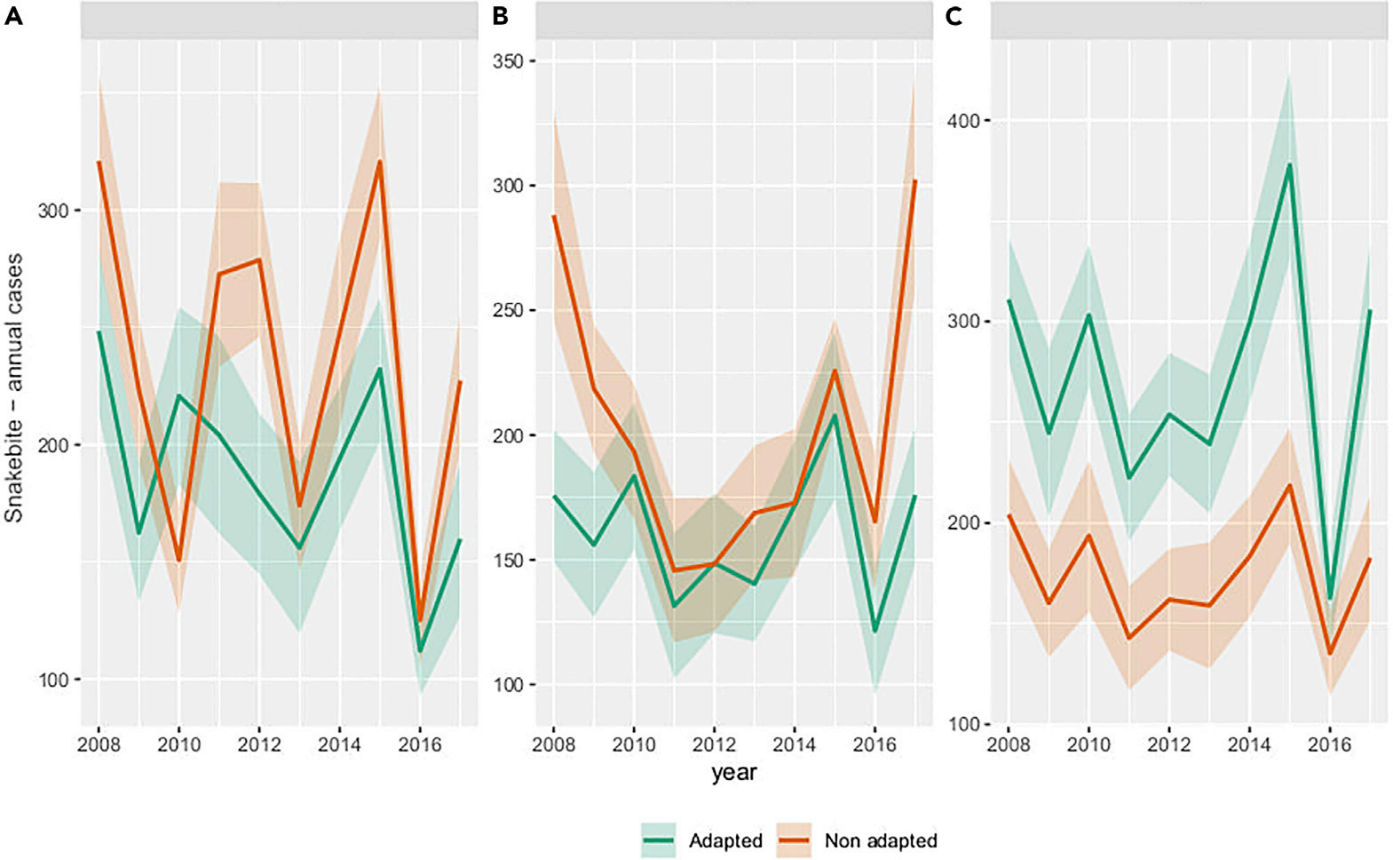
Annual snakebite cases (+/− 95% confidence interval) in response to the change in yearly precipitation for both adapted and non-adapted farmers (A) Variability in the number of bites for rice farmers resulted in years where adapted farmers were at higher risk than non-adapted farmers, such as 2010. (B) The number of bites for adapted rubber farmers tended to be higher, but during certain years such as 2012 and 2015 the number of bites was similar between the two. (C) For tea farmers, the number of bites was consistently higher for adapted farmers in comparison to non-adapted farmers.

**Figure 4:**
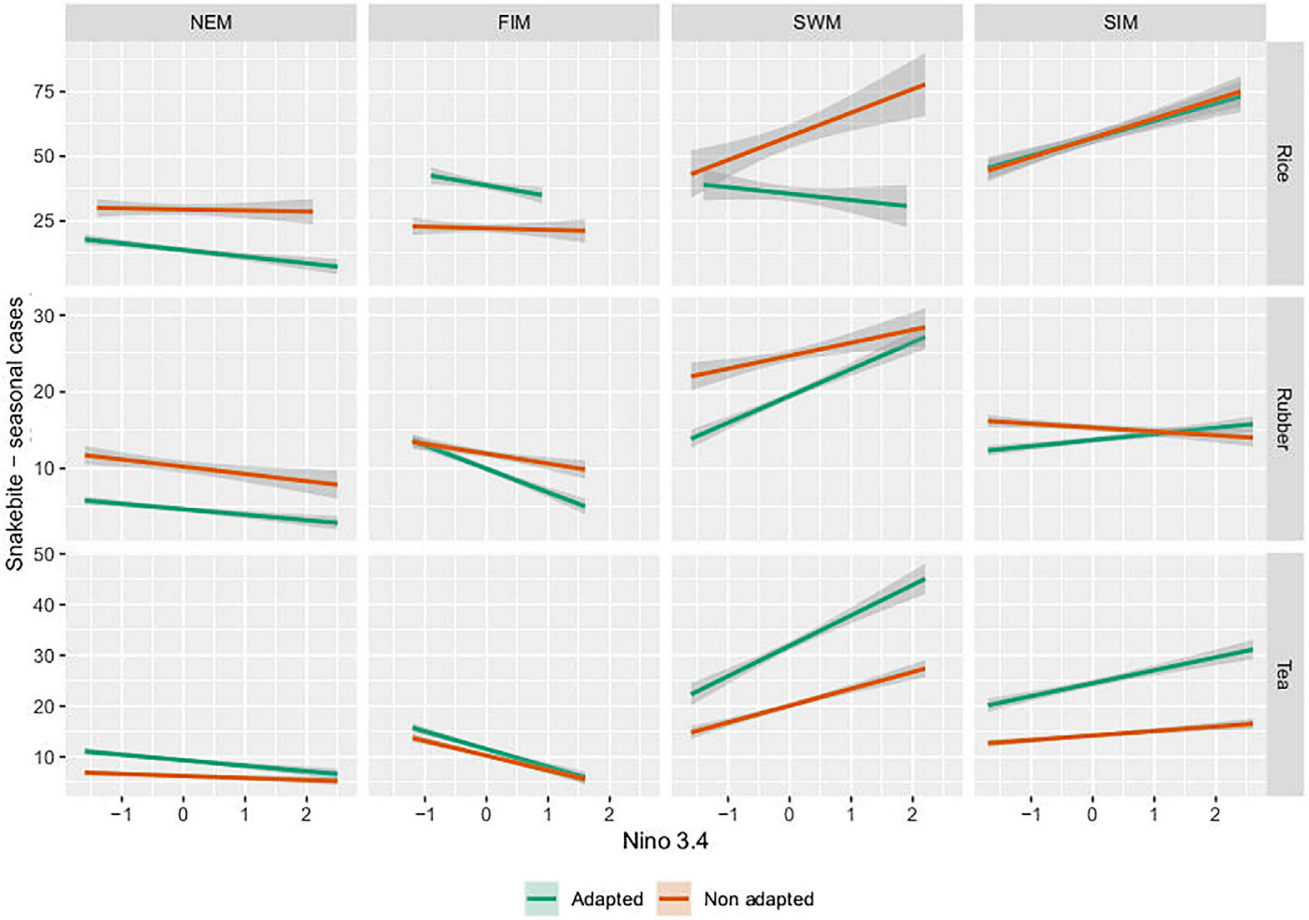
Linear regression analysis examining the effect of the NINO3.4 ENSO index on the total number of snake bites for each one of the four climate seasons FIM (March–April), SWM (May–September), SIM (October–November), NEM (December–February).

Changing the variety of rice cultivated in the Ratnapura region from regular long-duration paddy variety (3.5 months) to a drought-tolerant and short-duration paddy variety (2.5 months) decreased hourly per-capita snakebite incidence by an average 16% (99% CI 12–19%; p < 0.05) overall years (Figure 2A). There was also a 17% (99%CI 12–20%; p < 0.05) decrease in total snakebite count during the entire simulation period (Figure 2D). On closer inspection, these results were attributed to the greater overlap between farmer and snake activity periods under the baseline (long-duration paddy variety) compared to the adaptation (short duration) condition, leading to higher number of total snakebites and incidence. Nevertheless, there was also high annual variability in the difference between snakebites for adapted versus baseline strategies, which included some years in which agricultural adaptations increased snakebite risk (e.g., 2010) (Figure 3A). The reason for this variability was the sensitivity of the dynamics to the distribution of precipitation throughout the year and the dates of the southwest monsoon onset rather than yearly total amount of precipitation. This made rice farmers particularly susceptible to ENSO where positive and negative stages of the cycle tend to have divergent outcomes, particularly during the months of the southwest monsoon (Figures 3A and 4). This was the case during the southwest monsoon, which is a major rice farming period in the wet region. During this period, there was only an increase in snakebites for farmers in the baseline condition, which was affected by the NINO3.4 index (β_0_ = 57.7, β_1_ = 9.13, Adjusted R-squared = 0.02; Figure 4), whereas for farmers using adapted strategies the ENSO did not have any significant effect (p-value >0.05). This means that farmers who do not adapt are more susceptible to snakebite during El Nino events. Increased susceptibility is because of higher precipitation during the late Yala harvest season, which would affect farmers using non-adapted strategies more than those who use adapted strategies and harvested their short-duration paddy variety earlier. On the other hand, both adapted and non-adapted strategies showed susceptibility to El Nino events during the second intermonsoonal period (β_0_ = 56.9, β_1_ = 6.71, Adjusted R-squared = 0.1; β_0_ = 57.15, β_1_ = 7.4, Adjusted R-squared = 0.12, respectively) when farmers using both adapted and non-adapted strategies are planting rice fields for the Maha season.

### Rubber

Climate adaptation of the rubber harvesting method to low-frequency tapping increased hourly per-capita snakebite incidence compared to the baseline condition by an average 33% (99 CI 30–36%; p-value <0.05) across all years (Figure 2B). On the other hand, changing from traditional to low frequency tapping method decreased the total number of snakebites by 17% (99%CI 14–19%; p < 0.05) over the entire simulation period (Figure 2E). The reason for the increase in hourly per-capita snakebite incidence but not in number of bites because of adaptation was related to the ways labor is allocated in time, as well as the number of farmers working in the plantations. Although traditional farming approaches are associated with a lower number of workers during the months of May to August when snake activity is high, farmers who apply adaptive strategies continuously work during these months thereby increasing the risk per farmer. On the other hand, adaptive strategies mean that there are fewer farmers working in the plantations at any given moment, thereby decreasing the mean yearly number of bites overall but maintaining high risk for those farmers who are continuously working.

Differences in outcomes relating to the NINO3.4 index had to do with the combination of changes in number of rainy days as well as total amount of precipitation. An increase in total precipitation led to a rise in number of bites for both adapted and non-adapted strategies, whereas an increase in total rainy days reduced the number of bites when using non-adapted strategies because of loss of work days. During the north east monsoon, there was a negative effect of the NINO3.4 index and number of bites for farmers using adapted strategies (β_0_ = 4.69, β_1_ = −0.71, Adjusted R-squared = 0.07) and non-adapted strategies (β_0_ = 10.23, β_1_ = −0.93, Adjusted R-squared = 0.02), meaning the La Nina events led to an increase in snakebites, but the effect on number of bites for both groups was rather minor because this is a dry period in the Ratnapura region (Figure 4). During the first intermonsoon there was a negative effect of the NINO3.4 index on number of snakebites for farmers using both adaptive strategy (β_0_ = 9.96, β_1_ = −3.06, Adjusted R-squared = 0.14) and non-adapted strategy (β_0_ = 11.9, β_1_ = −1.28, Adjusted R-squared = 0.02) meaning that for both groups La Nina events led to an increase in snakebite during that season. On the other hand, during the Southwest monsoon there was a positive effect of the NINO3.4 index on number of snakebites, for farmers using both strategies (β_0_ = 19.47, β_1_ = 3.5, Adjusted R-squared = 0.06) and non-adapted strategies (β_0_ = 24.71, β_1_ = 1.68, Adjusted R-squared = 0.005), meaning that El Nino events led to increased numbers of snakebites for both groups. During the second intermonsoon there were opposite patterns between the adapted and non-adapted strategies, where farmers using adapted strategies showed a positive effect from the NINO3.4 index (β_0_ = 13.71, β_1_ = 0.8, Adjusted R-squared = 0.02) and non-adapted strategies a small negative effect (β_0_ = 15.33, β_1_ = −0.5, Adjusted R-squared = 0.005) (Figure 4). This opposite pattern was possibly caused by the interplay between number of rainy days and total amount of rain, whereby an El Nino event during this season would cause farmers using adaptive strategies to work under risky conditions, while preventing those using non-adaptive strategies from working in those same conditions because of rainy days that prevent them from harvesting rubber.

### Tea

Changing from continuous to optimized tea harvesting methods increased hourly per-capita snakebite incidence on average by 7% (99% CI 5–9%; p < 0.05; Figure 2C) and the total number of snakebites increased by 56% (99% CI 52–58%; p < 0.05; Figure 2F). There was an interannual variation in the difference between farmers using adapted and non-adapted strategies, but those using adapted strategies consistently had a higher number of snakebites each year (Figure 3C). The reason for the increase in both total number of bites and hourly per-capita snakebite incidence was related to the ways labor is allocated in time, as well as the number of farmers working in the plantations. The larger number of farmers using adapted strategies working during peak snake activity in the months of June–September because of increased tea yield during these months, when snakes are more active, lead to increased snakebite risk per capita for those same farmers.

Among farmers using adapted strategies there was a significant effect on the number of snakebites by the NINO3.4 index during all 4 seasons (Figure 4). The effect was strongest during the first intermonsoon when famers using either adapted strategies (β_0_ = 11.56, β_1_ = −3.6, Adjusted R-squared = 0.13) and non-adapted strategies (β_0_ = 10.28, β_1_ = −2.89, Adjusted R-squared = 0.12) both showed a negative effect from the index, meaning that La Nina events would cause an increase in snakebite for that period. There was also a significant positive effect during the southwest monsoon for both adapted strategies (β_0_ = 31.9, β_1_ = 5.99, Adjusted R-squared = 0.05) and non-adapted strategies (β_0_ = 20.13, β_1_ = 3.3, Adjusted R-squared = 0.04), meaning the El Nino events increase snakebites during that season.

## Discussion

Elevated greenhouse gas concentrations further intensify the impacts of ENSO in the tropics,^54^ causing more severe and frequent droughts and more intense flooding,^55^ as well as changes in the timing of monsoon onset.^56^ Increased intensity in climate cycles and its variability has considerable implications for human health, including enhanced morbidity and mortality because of heatwaves, changes in the distributions of zoonotic diseases, and via threats to food security such as crop failure.^60^^,^^62^ Changes in snakebite patterns and burden are partially because of snakes’ responses to climatic conditions, including seasonality, ENSO^4^^,^^11^ and spatial variability.^7^^,^^12^^,^^36^^,^^64^ Recent studies highlight socio-economic and behavioral factors of human populations, in particular farmers’ agricultural practices, as important drivers of snakebite.^4^^,^^36^^,^^57^ Yet, the impacts of climate change adaptation strategies by farmers in the tropical frontier regions on snakebite risk and burden remains unstudied. Based on previous research, we hypothesize that adaptation strategies could influence the contact rate between farmers and snakes, and that this could be further affected by extreme ENSO phases. Here we explore the effect of farmers’ adaptation strategies to climate change on the human-snake contact process, to predict how these adaptations may alter snakebite risk. We do this by using an agent-based simulation model based on empirical datasets on snake and farmer behavior in Sri Lanka, a snakebite hotspot.^19^ Our simulations are based on a simplified model in which farmers’ behaviors change, with snake activity patterns and behaviors. This approach allowed us to isolate the effect of farmers’ behaviors from other factors occurring in parallel, and to observe how this affects snakebite.

We found that unintended outcomes of farmers’ climate change adaptation strategies (e.g., use of a short-duration paddy variety) can include both increases or decreases in snakebite risk (bites per farmer per working hour, total number of annual bites) because of the changes in the human-snake contact process (Figure 2). Our results indicate that shifting to low frequency rubber tapping methods to adapt to a changing climate would lead to a 33% increase in average snakebite incidence per farmer per working hour, but a 17% decrease in the total number of snakebites in the entire study area because of decreased labor in plantations in general. Choosing a short-duration rice variety to adapt to a changing climate led to a 17% reduction in the average snakebite incidence per farmer per working hour by pushing forward the harvest season into months with less snake activity. In contrast, adaptation of tea harvest practices to a changing climate led to a 7% increase in snakebite incidence per farmer per working hour as well as a 56% increase in the total number of bites over the study area and across all years in the study window.

Of interest, we found that the key climatic factors such as monsoon onset dates and precipitation levels, as well as their correlation with ENSO, lead to different snakebite risks between crop and farmer types (Figure 4). Previous research has pointed out that both cold and hot phases of ENSO could increase snakebite risk.^4^ Here we have shown that this should be understood within the context of the agricultural practices used as well as the specific season affected by ENSO. For rice farmers using climate change adaptation strategies, there was a reduction in the number of snakebites associated with the timing of monsoon commencement and its length. Rice farmers who used climate change adaptation strategies showed lower risk particularly during the southwest monsoon when ENSO was in a positive phase (Figure 4) because the peak of the monsoon did not coincide with their harvest schedule. For rubber farming, major differences could be seen during the first intermonsoon, and the southwest monsoon, where ENSO-related higher precipitation had a stronger effect on farmers using climate change adaptation strategies (Figure 4). One reason for this was that those farmers who used adaptation strategies harvested rubber regardless of rainfall, meaning that they went out working more during risky months, whereas farmers who did not use adaptation strategies were less prone to work during those months and were therefore less affected by ENSO. Tea farmers using climate change adaptation strategies were particularly affected by ENSO during the southwest monsoon, and the second intermonsoon, because of increased labor demands that coincided with risky months. These diverse patterns show that the risk of health maladaptation depends not only on the specific farming strategy chosen by the farmer, but also on the interaction between these strategies and ENSO.

The possibility of unintended consequences of adapting farming practices highlights the importance of studying the risks associated with climate variability using a multi-risk approach^66^^,^^68^ that examines how different natural hazards are related to one another. This systems thinking approach^58^ could also help us better understand the multiple risks that rural farmers face under climate change,^71^^,^^73^ by linking social, economic, and climatic factors to daily life practices in ways that elucidate what makes specific social groups particularly vulnerable to snakebite, and other neglected (including vector-borne and zoonotic) tropical diseases. Previous research has illustrated how the presence of people at new points in time or space could generate new contact processes that were not previously possible.^59^ We have shown that in some cases this could lead to health costs when adaptation strategies related to climate change, representing a maladaptation for health. Equally, we have shown the reverse can occur, where the climate change adaptation strategy could instead reduce snake-human contact, thereby producing a health co-benefit or win-win (other potential costs notwithstanding, e.g., impacts to other infections or costs in other sectors) (Figure 2).

Rice farmers in Sri Lanka are increasingly using short-duration paddy varieties to avoid the negative impacts of the increasingly frequent droughts. Such a strategy tends to decrease the number of contacts and the number of snakebites (Figures 2A and 2D) resulting in a co-benefit. In some cases, greater complexity is predicted with the net effect on snakebite risk more nuanced; for instance, rubber farmers facing droughts may shift to low-frequency tapping methods, which would increase snakebite incidence per farmer by up to 33% (Figure 2B) but reduce the number of bites in total (Figure 2E) because of lower labor intensity in the plantations. We also found that optimization of tea harvest would increase contacts and total number of snakebites and incidence (Figures 2C and 2F) constituting another example of a climate change adaptation strategy being potentially maladaptive for human health. These findings call attention to the need for understanding health outcomes on multiple fronts. For example, subsistence farmers have been previously recognized as being at high risk of snakebite.^61^ Although these farmers are one of the populations most affected by climate change,^18^ many of them may not have the means to adapt to climate variability.^63^ For rice farmers in particular this may lead to a co-harm effect where those who cannot adapt will be at greater risk of both crop failure and snakebite (Figures 2A and 2D). This finding highlights the need to study all of the varied consequences of an individual adaptation strategy, and to avoid overgeneralization of future risks under climate change.

Although our simulation method is effective in modeling future scenarios, there are several limitations to our approach to modeling snakebites. Because our model represents only small-scale farmers’ behaviors in a single district of Sri Lanka, our results are not directly generalizable to other scales, and we recognize that our model only focuses on certain aspects of a subsystem embedded within a more complex system that ultimately determines patterns of human-snake interactions and snakebite at, for example, a national scale. Although the small scale interactions modeled in this study using ABM allow us to better understand how individual behavior contributes to snakebite epidemiology, it is still limited in reproducing macro dynamics because of limited computational power. At present, larger scale predictions of snakebite patterns,^65^ including under global change^20^ have been explored with other approaches tailored for these purposes, and have shown how large scale changes in snakebite patterns occur because of climate change using epidemiological models. These macro spatial and temporal models allow integration of snake distribution patterns into the models, allowing for increased abundance and local extinction under climate change, processes which have also been shown to have an effect on snakebite. In addition, our model represented only one change in human behavior, but in reality farmers may choose several behavioral adaptation strategies and other changes (e.g., technological) at once, leading to adverse effects on the human-snake encounter process. For instance, famers may change timing of cropping but also use PPE or mechanization. Furthermore, our use of past climate during a defined temporal window limits the predictability of our model. Future climate patterns would not only potentially influence the distribution of snakes, but also snake activity levels through time, leading to novel snakebite patterns. Finally, there was not enough data on location and percentage of farmers using adaptation methods to properly validate the results of our scenarios. Nevertheless, we intend for this modeling effort to shift focus to this issue and highlight the importance of gathering more information on farmer adaptations and their connections to health hazards including snakebite and other zoonotic and vector-borne diseases they may be exposed to.

Our approach to modeling snakebite risk because of climate change adaptation could similarly be applied to other zoonotic contact processes associated with agricultural activities, such as exposure to malaria transmitting mosquitoes in plantations, which is correlated with the utility density of different land covers (e.g., Fornace et al., 2019^75^), or exposure to non-human primates, which is associated with landscape and livelihood activities (e.g., Mcintosh and Lambin, 2020^76^). Although the study of climate change adaptations has been gaining momentum in the academic community,^67^^,^^69^^,^^70^ not enough attention has been given to the possibility of adverse consequences on health from adaptations in agricultural practices, and further studies are needed.^28^ Our results show the importance of studying each adaptation strategy individually, within the climatic and ecological context in which it is applied, to understand the potential risks of zoonotic contact processes associated with it. Furthermore, our results highlight the importance of collecting high quality biological data on snakes, which allows the development of integrative models that capture more closely the mechanisms involved in human-snake contact and the outcomes of those contacts. This should be central to efforts to prevent snakebite in particular, as called for in the WHO snakebite roadmap, but could serve as a model for other zoonotic diseases as well.

With ENSO expected to intensify under climate change and its subsequent effects on agriculture across South East Asia, farmers are expected to adapt to new conditions in a multitude of ways, including planting new crop varieties, changing the timing of planting seasons, and using new technologies.^17^^,^^24^^,^^42^^,^^45^^,^^72^ Because South East Asia, and the tropics in general, are some of the locations most susceptible to snakebite,^61^^,^^74^ understanding how farmer adaptation and snakebite are related is of utmost importance for both preventing snakebite and for understanding the potential harms caused by climate change or our reactions to reduce its impacts. In addition, understanding health risks associated with either adaptation or non-adaptation could help elucidate vulnerabilities of marginalized communities. Our model simulation provides new insights into the possible health maladaptations caused the adaptation strategies necessary to withstand the hazards posed by climate change and associated variability. The insights gained through these simulations could allow us to better focus our efforts to reduce snakebite burden in a rapidly changing world, as well as help achieve the WHO goal of reducing snakebite mortality by 50% by the year 2030.^29^

### Limitation of the study

Although our simulation method is effective in modeling future scenarios, there are several limitations to our approach to modeling snakebites. Because our model represents only small-scale farmers’ behaviors in a single district of Sri Lanka, our results are not directly generalizable to other scales, and we recognize that our model only focuses on certain aspects of a subsystem embedded within a more complex system that ultimately determines patterns of human-snake interactions and snakebite at, for example, a national scale. Although the small scale interactions modeled in this study using ABM allow us to better understand how individual behavior contributes to snakebite epidemiology, it is still limited in reproducing macro dynamics because of limited computational power. In addition, our model represented only one change in human behavior, but in reality farmers may choose several behavioral adaptation strategies and other changes (e.g., technological) at once, leading to adverse effects on the human-snake encounter process. For instance, famers may change timing of cropping but also use PPE or mechanization. Furthermore, our use of past climate during a defined temporal window limits the predictability of our model. Finally, there was not enough data on location and percentage of farmers using adaptation methods to properly validate the results of our scenarios.

## STAR★Methods

### Key resources table

**Table:** 

REAGENT or RESOURCE	SOURCE	IDENTIFIER
**Software and algorithms**		

Snakebite model	Goldstein et al. 2021	https://github.com/pogoyoly/snakebite_model

### Resource availability

#### Lead contact

Further information and requests for resources and reagents should be directed to and will be fulfilled by the lead contact, Eyal Goldstein (eyal.goldstein@forst.uni-goettingen.de).

#### Materials availability

DOIs are listed in the key resources table. Original input for the model is listed in Table 1.

### Method details

Our model includes two types of agents: farmers and snakes. Each farmer is an autonomous agent who works on a number of possible agricultural land cover types according to the scenario that is ascribed. Farmers have working schedules that include the land cover type they should be farming, time of day they begin to work, and the number of hours they will spend working in that land cover class. Using the work schedule, based on information gathered during field work, the farmers move between the land cover they are farming and their home.

Each snake agent is characterized by a set of ecological and behavioural traits, including: species, daily activity, habitat preference, propensity to bite, and seasonal activeness. Each species is given a set of probabilities of moving between land cover classes depending on their habitat preferences and the area covered by each land cover class. Seasonal activity of each snake species in the model is driven by precipitation patterns.

The purpose of the model is to represent the spatio-temporal overlap between farmers and snakes according to the prevailing weather (2008–2017), with the farmers reacting to longer term climatic and landscape conditions by adapting their farming practices. Both farmers and snakes seasonally interact with their climate and landscape, which define their behavioural patterns, and when they meet while being active, the model tracks the possibility of a snakebite occurring according to the different species’ propensities to bite. We then run the model according to different climate adaptation scenarios for each crop type, and follow the snakebite outcomes accordingly. For each scenario, we compared the outcomes of the no adaptation (baseline) vs adaptation strategies across all years.

For the rice farmers scenario, we tested whether the use of a short-duration paddy variety would affect snakebite risk. Farmers in the simulation are allowed to plant either a conventional variety with three months growth period and harvest in the fourth month, or a drought-resistant variety with two months growth period and harvest at the third month. We focus on the differences in snakebite risk under years with different precipitation patterns that reflect the impacts of ENSO.

For rubber famers we simulated different rubber-tapping methods and compared snakebite risk between the current practice of farmers continuously tapping rubber trees on non-rainy days leading to approximately 150 workdays per year, and farmers implementing the adaptation strategy of low frequency tapping, where rubber trees are tapped only once every five days irrespective of rainfall, through the entire year.

For tea farmers we developed a tea-harvest scenario in which we compared current tea harvesting strategies and an optimised tea harvest strategy that predicts and meets labour requirements on the basis of the linear relationship between yield and rainfall in the month prior to optimal rainfall, with a work increase of 0.514 labour hours per hectare above the baseline for every mm of rainfall.

## Data Availability

All original code has been deposited at https://github.com/pogoyoly/snakebite_model and is publicly available as of the date of publication. Any additional information is available from the lead contact upon request.
