# Supplementary material for: Climate change maladaptation for health: Agricultural practice against shifting seasonal rainfall affects snakebite risk for farmers in the tropics
Source: iScience. 2023 Jan 7;26(2):105946. doi: 10.1016/j.isci.2023.105946 (PMC9932500; doi:10.1016/j.isci.2023.105946)

iScience, Volume *26*

Supplemental information

Climate change maladaptation for health: Agricultural practice against shifting seasonal rainfall affects snakebite risk for farmers in the tropics

Eyal Goldstein, Joseph J. Erinjery, Gerardo Martin, Anuradhani Kasturiratne, Dileepa Senajith Ediriweera, Ruchira Somaweera, Hithanadura Janaka de Silva, Peter Diggle, David G. Lalloo, Kris A. Murray, and Takuya Iwamura

Supplamentary material

S1

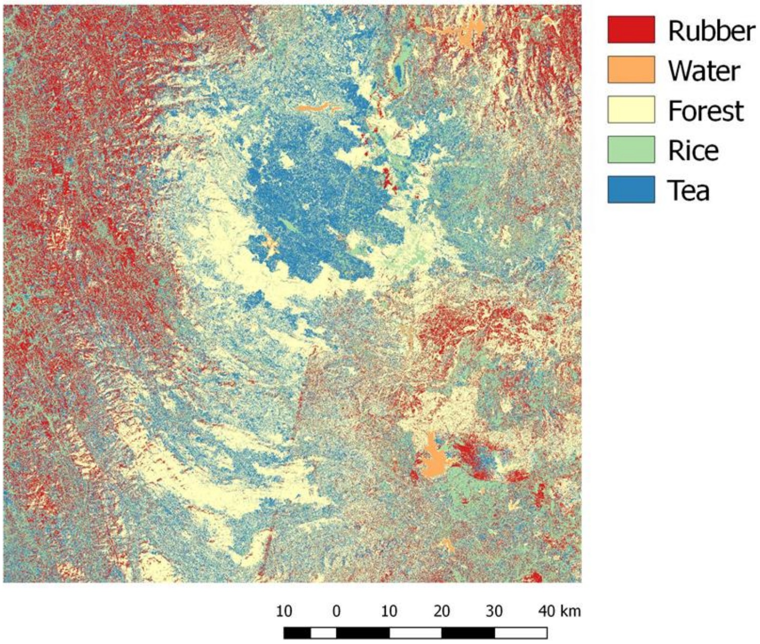

Supplement: Document S1. Supplementary material 1 [file mmc1.pdf]
